# Effectiveness of Lee Silverman Voice Treatment® LOUD on Japanese-Speaking Patients with Parkinson's Disease

**DOI:** 10.1155/2020/6585264

**Published:** 2020-01-24

**Authors:** Keigo Nakayama, Toshiyuki Yamamoto, Chihiro Oda, Masako Sato, Takeshi Murakami, Satoshi Horiguchi

**Affiliations:** ^1^Department of Rehabilitation Medicine, National Center Hospital, National Center of Neurology and Psychiatry, 4-1-1 Ogawahigashi, Kodaira, Tokyo, Japan; ^2^Speech-Language and Hearing Science, Kitasato University Graduate School of Medical Sciences, 1-15-1 Kitasato, Minami-ku, Sagamihara, Kanagawa, Japan; ^3^Department of Neurology, National Center Hospital, National Center of Neurology and Psychiatry, 4-1-1 Ogawahigashi, Kodaira, Tokyo, Japan

## Abstract

*Background. *Lee Silverman Voice Treatment® LOUD (LSVT®) is an intensive program devised in the United States to train patients with Parkinson's disease (PD) to speak louder, at normal intensity, while keeping a good voice quality. Four weeks of LSVT® has been shown to increase vocal loudness and improve intelligibility among Japanese-speaking PD patients. However, the long-term effects of LSVT® have not been examined in these patients.* Objective*. This study aimed to investigate the long-term effects of LSVT® on Japanese-speaking PD patients.* Methods*. Twenty-one Japanese PD patients underwent a standardized course (four sessions over four consecutive days, for four weeks) of LSVT® at our hospital. Vocal loudness and intelligibility were assessed at the following three time-points: pretreatment (baseline), immediately after treatment, and at the end of the 12 month follow-up (12FU). Sound pressure levels (dB SPL) were measured during the following tasks: sustained phonation of /a/, reading a standardized text, and delivery of a monologue. Three experienced speech-language pathologists, who were blinded to patients' identities and assessment points, assessed speech intelligibility based on recorded audio samples of each participant during the reading and monologue tasks. *Results*. Fourteen patients were evaluated at 12FU. Changes in dB SPL from baseline to immediately after treatment were +6.5 dB, +4.2 dB, and +2.8 dB, and those from baseline until 12FU were +4.7 dB, +3.5 dB, and +2.5 dB in sustained phonation of /a/, reading a passage, and delivery of a monologue, respectively. These changes were significant (*p* < 0.025) in both the baseline-to-immediately-after-treatment and baseline-to-12FU intervals. Intelligibility relative to baseline was significantly improved immediately after treatment, but not at 12FU. *Conclusions*. LSVT® had a long-term effect on the vocal loudness of Japanese-speaking PD patients. A short-term effect was seen in intelligibility, however, there was no significant long-term effect.

## 1. Introduction

Approximately 90% of Parkinson's disease (PD) patients present with dysarthria, characterized by reduced vocal loudness, reduced intonation variability, hoarseness, increased speech rates, and improper articulation of consonants [1]. Reduced vocal loudness in PD patients is caused by reduction in subglottal pressure, expiratory flow, and vocal fold adduction [2]. Additionally, because of sensorimotor dysfunction, hypophonic PD patients perceive their voices as being of normal volume [3].

Lee Silverman Voice Treatment® LOUD (LSVT®) is an intensive program devised in the United States to train patients with PD to speak louder, at normal intensity, while keeping a good voice quality [4]. The program is in accordance with motor learning theories that focus on specificity, intensity, repetition, and saliency; LSVT® focuses solely on vocal loudness, which improves by repeated training on a single target with a maximum effort [5]. In addition, LSVT® trains patients to recognize how loudly they must speak to reach a normal volume [3]. If patients can identify the volume of their voice, the effects of LSVT® are maintained after training [6]. After PD patients are trained to speak more loudly and to use the louder voice in daily life, they can maintain increased vocal loudness long-term [7]. A randomized, controlled trial of LSVT® in English-speaking individuals with PD reports significant improvement in the sound pressure level (dB SPL) [8]. A long-term follow-up study reports a significant increase in vocal loudness two years after LSVT® [9]. In addition, LSVT® is said to have long-term effects compared to other training methods [10]. There are reports that LSVT® has improved intelligibility; in other cases, intelligibility has not changed or decreased [11].

English speaking PD patients with hypokinetic dysarthria have the same salient speech features as PD patients who speak other languages, such as Spanish and Korean [12]. Japanese-speaking PD patients also present with dysarthria and have the same speech characteristics as English-speaking patients [13]. Studies on LSVT® have been conducted in a few other languages and have primarily focused on the analysis of acoustic parameters before and after treatment [14]. The increase in conversational intelligibility in both English and Spanish dysarthria in PD patients following LSVT® might be explained by language-universal factors [15]. Despite the fact that the Japanese language system uses the mora and the syllable is used in English [16], LSVT® for four weeks has been demonstrated to increase vocal loudness and improve intelligibility in Japanese-speaking PD patients [17]. However, it is not clear whether LSVT® has long-term effects on vocal loudness and intelligibility in different language systems. Therefore, the present study examined the long-term effects of LSVT® on Japanese-speaking PD patients.

## 2. Materials and Methods

### 2.1. Subjects

This study included 21 Japanese PD patients (13 men and eight women; median age: 68.0 years [range: 64.0−70.5 years]; median disease duration: 9.0 years [range: 4.0–12.5 years]; median Modified Unified Parkinson's Disease Rating Scale (MDS-UPDRS): 37.0 [range: 24.5–52.0]; median MDS-UPDRS Part II-1 (Speech): 1.0 [range: 1.0–2.0]; median MDS-UPDRS Part III-1 (Speech): 1.0 [range: 1.0–2.0]; median Hoehn & Yahr Scale stage: 3.0 [range: 2.0−3.0]; median Raven's Colored Progressive Matrices score: 32.0 [range: 29.5–34.0]. The patients learned about the implementation of LSVT®through our internet and television campaigns; they came to the hospital hoping to receive LSVT® training. They underwent LSVT® at our hospital between April 2011 and February 2018 (Table 1). All patients were clinically diagnosed with possible PD because they met the International Parkinson and Movement Disorder Society clinical diagnosis criteria [18]. Patient No. 4 was excluded because it was juvenile PD. In addition, dopamine transporter single-photon emission coupled tomography revealed striatonigral degeneration, and metaiodobenzylguanidine myocardial scintigraphy found a reduced heart-to-mediastinum uptake ratio (delayed images) in all patients. Treatment with antiparkinsonian drugs improved motor symptoms in all patients. Patients with the following conditions were excluded from the study: cerebrovascular disease (as indicated by head magnetic resonance imaging) that could result in dysarthria, previously diagnosed laryngeal disorders, hearing-impairments that interfered with communication in everyday conversation, and dementia resulting in difficulty with the LSVT® program.

The study was approved by our hospital's institutional review board (approval no. 2016-013). The study used training and test data that were collected in routine clinical practices. Acquisition of new data was not conducted in this study. Therefore, informed consent was considered unnecessary. The information needed was advertised on the Ethics Committee's website of the National Center of Neurology and Psychiatry; the participant had an opportunity to refuse to participate in this study.

### 2.2. Assessment Methods

Three LSVT® globally-certified speech-language pathologists performed the LSVT® on all patients. In accordance with the LSVT® program, personalized training was conducted for four consecutive days per week (60 min per day) for four weeks. During each therapy session, patients practiced daily exercises including: maximum sustained phonation of “ah”, maximum fundamental frequency range, and reading of functional phrases. During training, patients were constantly made aware of speaking loudly and underwent sustained phonation practice, vocal range training, and conversation practice [6]. All patients received daily homework and carry-over tasks, with the recommendation to focus on “speak LOUD”. However, it was taught not to shout. The dosages of antiparkinsonian drugs were not changed for these patients during the study period. Patients did not undergo any other forms of speech therapy.

All assessments were conducted independently of the trained SLP. The assessments described below were conducted at the following times: (1) pretreatment (baseline), (2) immediately after four weeks of treatment, (3) at the end of the 12 month follow-up (12FU) after four weeks of treatment. “Follow-up” was only evaluated at the outpatient department of neurology. LSVT® encourages self-training in daily life after the end of training. Monitoring self-training by phone, etc. is recommended, but in Japan it is often difficult to continuously check the effect after the end of LSVT®. Therefore, we have not included details on the number of times self-training was conducted. In this study, the follow-up evaluation was conducted 12 months after training.

### 2.3. Vocal Loudness

Vocal loudness tests, which were conducted in a soundproof room, consisted of the following items. (A) Sustained phonation of /a/. The test administrator instructed the patient to take a deep breath and make the sound /a/ at a normal volume for as long as possible. The task was performed six times; the mean phonation time was used as the measured value. (B) Reading a passage: “The North Wind and the Sun”. The Assessment of Motor Speech for Dysarthria (AMSD) was used in this task. The font size was enlarged to A3 paper size and placed at the patient's eye level. The patient was then instructed to read the passage in Japanese aloud at a normal volume. (C) Delivering a monologue: this task used the Japanese folk tale “Momotaro (The Peach Boy)”. The patient was instructed to talk about the story at a normal volume for one min.

During vocal loudness tests, a calibrated sound level meter (NL-21, RION) was placed 30 cm from the patients' lips to measure SPLs. SPLs, displayed every second, were summed up; the total was divided by the displayed time (s) to obtain the patient's mean vocal loudness. Noise level of 60 dB or less was regarded as background noise. The background noise part was regarded as a pause portion. The measurement was made by excluding the pause portion associated with reading aloud. A recorder (PCM-D50, SONY, Tokyo) was placed 30 cm from the patients' lips to record his or her voice during all tasks.

### 2.4. Intelligibility

Intelligibility was assessed based on reading of a passage and production of a monologue, recorded in vocal loudness testing. Patients were recorded before treatment, immediately after treatment, and at 12FU. The audio samples were arranged randomly and assessed at a constant SPL by three speech-language pathologists, with at least five years of experience. Listeners were encouraged to listen to the recorded audio at a loudness of 60% on the speakers of a personal computer (windows8 and later) in a quiet space. The audio data were copied and reassessed to examine the reproducibility of the original assessments. A 9-point Likert scale may be used to evaluate speech intelligibility [19]. Intelligibility was assessed using a standard Japanese test, as described by Itoh [20]. Audio samples were assessed on a nine-level scale in 0.5-point increments, including: “1. Completely intelligible,” “2. Some unintelligible words,” “3. Intelligible if the listener knows the topic,” “4. Few intelligible words,” “5. Completely unintelligible”. Intraclass correlation coefficients (*ICCs*) were calculated to determine the assessor with the highest reliability; the assessor's assessments of intelligibility were used as reference values.

### 2.5. Statistical Analyses

Vocal loudness and intelligibility were compared using the Friedman test. Differences between baseline and immediately after treatment, and between baseline and at 12FU were compared using the Wilcoxon signed-rank test. In addition, the discontinued follow-up group and the follow-up group were compared by the Wilcoxon signed-rank test. Because of the potential effects of repeated measures, a *p-value of <0.025* (based on Bonferroni's inequality) was considered significant. Statistical analyses were performed using IBM SPSS® Statistics (ver. 25).

## 3. Results

**Figure 1:**
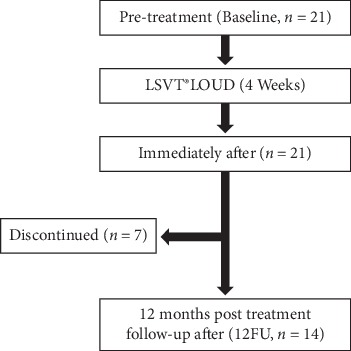
Follow-up schedule. LSVT®: Lee Silverman Voice Treatment; 12FU: 12 month post treatment follow-up.

Among the 21 patients, 14 (66.6%) completed the 12FU evaluations (Figure 1). Seven patients discontinued follow-up visits during the study period because of the following reasons: changes to training to control speech rate, such as the tapping or Delayed Auditory Feedback methods (*n* = 3; 42.9%), strong feeling of fatigue when talking for long periods(*n* = 1; 14.3%), unaware of the effects (discontinued due to not coming to follow-up) (*n* = 1; 14.3%), difficulty in continuing to visit the hospital because of exacerbation of PD symptoms (*n* = 1; 14.3%), unknown reasons (*n* = 1; 14.3%).

### 3.1. Follow-Up Group vs. Discontinued Follow-Up Group

No significant differences in baseline characteristics were found between the follow-up group and the discontinued follow-up group (Table 2).

### 3.2. Vocal Loudness

**Figure 2:**
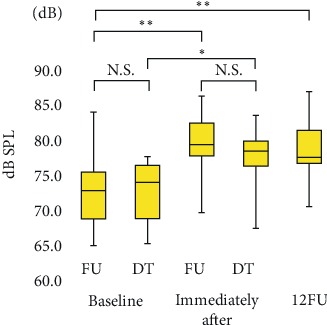
Sustained phonation of /a/.

**Figure 3:**
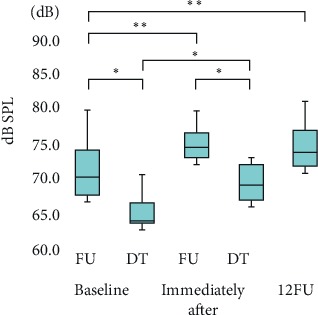
Reading a passage.

**Figure 4:**
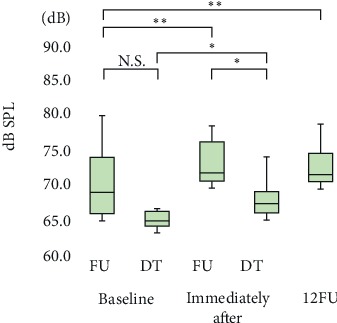
Delivery of a monologue.

Significant increase in vocal loudness (*p* < 0.01) in the sustained phonation of /a/ (+4.7 dB), reading of a passage (+3.5 dB), and delivering a monologue (+2.5 dB) were observed immediately after LSVT® treatment compared with baseline; the improvements persisted until 12FU (Figures 2–4). Notably, significant improvements (*p* < 0.05) in all the tasks were observed immediately after LSVT® treatment compared with baseline, even in the discontinued follow-up group (expect monologue).

Variability in dB was significant at all assessment points (*p* < 0.01). The median vocal loudness (interquartile range) values at: (1) baseline (FU, DT), (2) immediately after treatment (FU, DT), and (3) 12FU were : (1) FU: 72.8 (68.8−75.4) dB, DT: 73.9 (68.8-76.4), (2) FU: 79.3 (77.7−82.4) dB, DT: 78.5 (76.3-79.8), and (3) 77.5 (76.6−81.4) dB, respectively. Vocal loudness significantly improved immediately after treatment and at 12FU, compared with baseline. No significant difference in sustained phonation of /a/ was found between the follow-up group and the discontinued follow-up group. ∗*p* < 0.05; ∗∗*p* < 0.01*; N.S.: no significant difference*; FU: follow-up group; DT: discontinued follow-up group; 12FU: 12 month post treatment follow-up.

Variability in dB was significant at all assessment points (*p* < 0.01). The median vocal loudness (interquartile range) values at: (1) baseline (FU, DT), (2) immediately after treatment (FU, DT), and (3) 12FU were: (1) FU: 70.2 (67.6–74.0) dB, DT: 64.0 (63.6–66.4), (2) FU: 74.4 (73.0–76.4) dB, DT: 69.1 (66.9–71.9), and (3) 73.7 (71.7–76.9) dB, respectively. Vocal loudness significantly improved immediately after treatment and at 12FU, compared with baseline. There was a significant difference in reading a passage between the follow-up group and the discontinued follow-up group (*p* < 0.05). ∗*p* < 0.05; ∗∗*p* < 0.01.

Variability in dB was significant at all assessment points (*p* < 0.01). The median vocal loudness (interquartile range) values at: (1) baseline (FU, DT), (2) immediately after treatment (FU, DT), and (3) 12FU were: (1) FU: 68.8 (65.8–73.7) dB, DT: 64.7 (64.1–66.1), (2) FU: 71.6 (70.4–75.9) dB, DT: 67.2 (65.9–68.9), and (3) 71.3 (70.3–74.4) dB, respectively. Vocal loudness significantly improved immediately after treatment and at 12FU, compared with baseline.

There was a significant difference in delivery of a monologue between the follow-up group and the discontinued follow-up group at immediately after treatment (*p* < 0.05). ∗*p* < 0.05; ∗∗*p* < 0.01*; N.S.: no significant difference*.

### 3.3. Intelligibility

**Figure 5:**
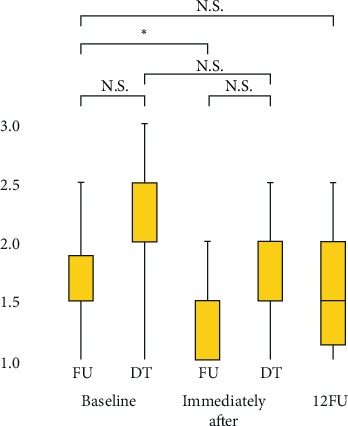
Intelligibility.

The ICC (*2, 1*) was *0.884*(*p* < 0.01) in the interrater reliability among the three assessors. The ICCs (*1, 1*) were *0.713*, *0.757*, and *0.809* (*all p* < 0.01) in the intrarater reliability of the three assessors, indicating significantly high reliability (Figure 5).

Variability in intelligibility was significant at all intelligibility assessment points (*p* < 0.05). 1: Completely intelligible; 2: Some unintelligible words, 3: Intelligible if the listener knows the topic. The median intelligibility (interquartile range) at (1) baseline (FU, DT), (2) immediately after treatment (FU, DT), and (3) 12FU were (1) FU: 1.5/5 (1.5–1.9), DT: 2.5 (2.0–2.5), (2) FU: 1.0/5 (1.0–1.5), DT: 2.0/5 (1.5–2.0) and (3) 1.5/5 (1.1–2.0), respectively. Intelligibility significantly improved immediately after treatment compared with baseline, but no significant improvement was observed at 12FU. No significant difference in intelligibility was found between the follow-up group and the discontinued follow-up group. ∗*p* < 0.025*; N.S.: no significant difference*.

## 4. Discussion

### 4.1. Vocal Loudness

In a previous study conducted in 21 PD patients in the United States, LSVT® significantly increased vocal loudness in: sustained“AH” phonation, reading of the “Rainbow Passage,” and conversational speech (monologue), immediately after treatment and at 24 months after treatment [8]. In a study conducted with Japanese-speaking PD patients, LSVT® significantly increased vocal loudness in reading aloud and delivering a monologue immediately after intensive training [17]. In the present study, Japanese-speaking PD patients showed a significant increase in vocal loudness immediately after treatment compared with before treatment; a significant increase in vocal loudness was also observed in these patients at 12FU. These findings indicate that LSVT® can maintain increased vocal loudness in Japanese-speaking PD patients for at least 12 months.

The long-standing theory is that the language symptoms of PD patients are movement disorders, due to hypoxia from muscle spasm and dopamine deficiency. However, in addition to movement disorders, sensory and neuropsychological factors are considered to be deeply involved. The hypothesis is that the following four abnormalities existing in the neural mechanism cause the problem of speech production: (1) scaling movement amplitude abnormality, (2) sensory processing abnormality, (3) internal cueing abnormality, (4) vocal vigilance abnormality. LSVT® is based upon elements derived from neurology, physiology, motor learning, muscle training, and neuropsychology. The five essential concepts of the LSVT® include: (1) focus on voice, (2) calibration, (3) high effort, (4) intensity, (5) quantify treatment related changes [21].

PD related dysarthria is very consistent among patients and can be interpreted and examined as a result of specific degeneration of specific neuronal populations. In particular, the treatment of loudness and dysprosody focused on the “universal language” aspect. It was considered to be a factor that showed therapeutic effects even if different languages were used [15]. On the other hand, PD dysarthria has been reported to be affected by the complexity of word initials, syllable-sized words, and changes in meaning due to stress accents; further investigation is necessary.

### 4.2. Intelligibility

A study conducted in “American English” demonstrated a significant improvement in vocal loudness and intelligibility after rate and loudness manipulations [22]. Similar findings have been obtained in Spanish speakers with PD [19]. Loud speech has been hypothesized to enhance cues to syllabic stress in English dysarthria (e.g., increased vocal intensity, improved pitch, and changed vowel articulation), thus potentially facilitating lexical stress segmentation [23]. In LSVT®, intelligibility is improved by not only increasing vocal loudness, but also improving intonation variability and reducing the speech rate [24]. A Japanese study with a nine-point scale also demonstrated a significant improvement in intelligibility after four weeks of training with LSVT® [17].

While LSVT® has been demonstrated to increase vocal loudness significantly, it did not significantly improve intelligibility (assessed by reading a passage or delivering a monologue, using a visual analog scale) [25]. In another study, 75% of subjects showed a significant improvement in intelligibility, but the remaining 25% showed either no change or a decline in intelligibility after LSVT®[11]. In addition, hypokinetic dysarthria associated with PD tends to worsen as PD progresses [26].

The present study found a significant short-term improvement in intelligibility following four weeks of training. The findings are similar to those in previous studies conducted in other countries or Japan. However, vocal loudness had no long-term effects on intelligibility. The decline in intelligibility at the 12FU may be due to the exacerbation of dysarthria over time. Factors other than vocal loudness, i.e., hoarseness and articulatory movements, may also have played a role.

### 4.3. Follow Up

In the United States, follow-up for LSVT® is difficult mainly because of geographical issues (being far away from institutions where LSVT® is performed) and difficulty in visiting a hospital due to movement disorders or the absence of a caregiver [27]. To address these problems, training methods are being developed and applied for patients living in remote areas; these methods have been demonstrated to be as effective as face-to-face training [28]. In this study, there was no significant difference in physical functions such as HY and MDS-UPDRS total between the continuation group and the discontinuation group. We think that this indicates the possibility that LSVT® can be continued even if the physical function is degraded. On the other hand, it was shown that even if the physical function is normal, the LSVT® may need to be interrupted. To facilitate long-term LSVT®, further studies are needed to improve the follow-up systems for patients in Japan who have the same difficulty in visiting a hospital as patients in the United States. To promote long-term LSVT®, it is first necessary to improve the follow-up system for Japanese patients who have difficulty visiting hospitals. In addition, further research is needed to measure mental and cognitive functions for people who maintain physical functions.

### 4.4. Limitations and Directions for Future Studies

This study showed short-term improvement in vocal loudness and intelligibility, but a long-term improvement was not observed in intelligibility after LSVT®. Another study with a small sample size found that intelligibility was difficult to be assessed in LSVT® [27]. The lack of a significant difference between 12FU and immediately after treatment may be due to the small sample size in the present study. Therefore, future studies are needed to investigate the effects of LSVT® with a larger sample of participants, and a number of more homogeneous severity subgroups. For example, we think it is necessary to obtain frequent evaluations, and self-training that captures subjective symptoms of speech, such as Voice Handicap Index (VHI). In addition, future studies are also needed to investigate the speech rush and the degree of hoarseness [24]. In this study, the follow-up period was 12 months; further studies are necessary to examine whether the effects last longer.

## 5. Conclusions

In conclusion, although the syllable structure of the Japanese language is different from that of English, LSVT® was shown to increase vocal loudness in the short and long term in Japanese-speaking PD patients. The exacerbation of motor symptoms forced some patients to discontinue LSVT®. Therefore, methods to ensure improvement in follow-up systems should be implemented for such patients in Japan. In addition, these results help show how LSVT® is performed in hospitals and clinics, and the duration for which its effects last in patients that are difficult to follow-up after training.

## Figures and Tables

**Table 1:** Patient characteristics.

Patients	Age (years)	Sex	PD duration (years)	MDS-UPDRS Total (ON)	MDS-UPDRS Part II-1 (speech)	MDS-UPDRS Part III-1 (speech)	HY	Raven CPM
1	52	F	4	32	1	1	3	34
2	71	M	25	43	2	2	3	30
3	77	M	2	23	1	2	3	32
5	68	M	9	44	1	1	2	32
6	65	F	5	40	2	2	3	34
7	66	M	9	37	1	2	3	34
8	68	M	5	40	2	2	2	30
9	70	M	19	74	1	1	3	34
10	69	M	13	78	4	4	3	20
11	51	F	3	33	3	1	2	35
12	60	F	3	30	2	2	3	28
13	63	M	8	26	0	0	2	36
14	74	F	13	18	0	0	2	30
15	73	F	3	18	1	1	1	35
16	67	F	10	15	1	1	1	29
17	68	M	12	57	3	3	3	27
18	55	M	5	18	1	2	3	29
19	66	M	9	47	0	2	3	32
20	85	F	10	57	1	1	4	32
21	65	M	15	62	2	1	2	30
22	68	M	4	26	3	1	2	30

The patients are listed in order of participation in the study. M: male; F: female; PD: Parkinson's disease; MDS-UPDRS: Modified Unified Parkinson's Disease Rating Scale; HY: Hoehn & Yahr Scale stage; CPM: Colored Progressive Matrices.

**Table 2:** Baseline characteristics in the follow-up group and the discontinued follow-up group.

	Follow-up group (*n* = 14)	Discontinued follow-up group (*n* = 7)	*Z*	*p*
Age	67.5 (64.5–69.3)	68.0 (55.0–77.0)	−1.183	0.237
PD duration	8.5 (3.8–13.0)	9.0 (4.0–10.0)	−0.508	0.611
MDS-UPDRS Total (ON)	36.5 (24.0–58.3)	37.0 (23.0–44.0)	−1.014	0.310
MDS-UPDRS	2.0 (0.8–3.0)	1.0 (1.0–1.0)	−1.511	0.131
Part II-1				
MDS-UPDRS	1.0 (1.0–2.0)	2.0 (1.0–2.0)	0.000	1.000
Part III-1				
HY	2.0 (2.0–3.0)	3.0 (3.0–3.0)	−1.134	0.257
Raven CPM	32.5 (28.8–35.0)	32.0 (30.0–34.0)	−0.271	0.786

PD: Parkinson's disease; MDS-UPDRS: Modified Unified Parkinson's Disease Rating Scale; HY: Hoehn & Yahr Scale stage; CPM: Colored Progressive Matrices.

## Data Availability

The data used to support the findings of this study are available from the corresponding author upon request.
